# Effect of aqua-cycling on pain and physical functioning compared with usual care in patients with knee osteoarthritis: study protocol of a randomised controlled trial

**DOI:** 10.1186/s12891-016-0939-5

**Published:** 2016-02-18

**Authors:** Stefanie Rewald, Ilse Mesters, A. F. Lenssen, Pieter J. Emans, Wiel Wijnen, Rob A. de Bie

**Affiliations:** Department of Epidemiology, CAPHRI School for Public Health and Primary Care, Maastricht University, Maastricht, The Netherlands; Department of Physiotherapy, Maastricht University Medical Centre+, Maastricht, The Netherlands; Department of Orthopaedic Surgery, Maastricht University Medical Centre+, Maastricht, The Netherlands

**Keywords:** Osteoarthritis, Aquatic exercise, Aqua-cycling, Immersed cycling, Underwater cycle ergometer

## Abstract

**Background:**

Over the last decade aquatic exercise has become more and more popular. One of the latest trends is aqua-cycling, where participants sit on a water-resistant stationary bike and, while immersed chest deep in the water, combine continuous cycling with upper body exercises that utilise water resistance. Since stationary cycling and aquatic exercises are frequently recommended to patients with knee osteoarthritis, combining both would seem an obvious step, and an aqua-cycling exercise programme for patients with knee osteoarthritis has indeed been developed. This study protocol gives a detailed description of the exercise programme and the methodology of a study to compare this programme with treatment involving usual care only.

**Methods:**

The study is a single-blind, parallel-group, randomised controlled trial of Maastricht University Medical Centre+, the Netherlands. Inclusion criteria: knee pain of four to seven on a 10-point pain rating scale; a Kellgren/Lawrence score between one to three; ability to cycle; good mental health; sufficient language skills; indication for physical therapy in conjunction with impairments due to OA. Exclusion criteria: any contra-indication for aquatic exercise; planned total knee replacement; corticosteroid injection <3 months and/or hyaluronic acid injection <6 months; severe joint complaints (other than knee joint); symptomatic and radiological apparent hip OA; inflammatory joint diseases; inability to safely enter and exit the pool; fear of water. Participants will receive two 45-min moderate intense aqua-cycling sessions weekly over a period of 12 weeks in addition to usual care or usual care only. Usual care consists of an individual intervention plan comprising lifestyle recommendations, medication routine and referral to a physical therapist. Participants will be assessed at baseline, and at 12 and 24 weeks after baseline. The primary outcome is self-reported knee pain and physical functioning. Secondary outcomes are lower limb muscle strength, functional capacity, self-reported disease severity, physical activity level, quality of life, self-efficacy and fear of movement. Daily diaries will collect information on knee pain, physical functioning, level of physical activity, pain medication routine and physical therapy (control group only) or exercise participation over two 30-day periods (during the intervention period).

**Discussion:**

To our knowledge the present study is the first randomised controlled trial evaluating the effects of aqua-cycling in the pre-surgical stage of knee osteoarthritis. This trial will demonstrate if the newly designed aqua-cycling intervention, in supplement to usual care, can help to improve impairments due to knee osteoarthritis.

**Trial registration:**

Netherlands Trial Register NTR3766 (21-12-2012).

**Electronic supplementary material:**

The online version of this article (doi:10.1186/s12891-016-0939-5) contains supplementary material, which is available to authorized users.

## Background

Aqua-cycling, which is cycling on a water-resistant stationary bike, might be a supplement to the available exercise possibilities for patients with knee osteoarthritis (OA). Knee OA, a common chronic health condition, affects the daily lives of millions of people worldwide by causing knee pain and difficulty performing day-to-day activities [1]. All dimensions of physical function, as described by the International Classification of Functioning, Disability, and Health (IFC) framework, are affected by knee OA [2]. For example, as a reaction to load-dependent joint pain that commonly occurs during daily functional activities like walking or stair-climbing, people tend to underuse the knee and become physically inactive [3, 4]. Avoidance of these activities gives rise to problems with body functions and structures such as cardiovascular deconditioning, muscle weakness and reduced knee range of motion, but also to more general health problems such as a higher risk of comorbidity and premature mortality [4, 5]. Exercise therapy is crucial for maintaining good general health and alleviating the symptom progression of knee OA [4, 6]. In addition to exercise, patient education about treatment options, weight management and strategies to prevent capacity overload of the damaged knee, as well as pharmacological treatment with analgesics or non-steroidal anti-inflammatory drugs (NSAIDs), are recommended for optimal conservative management of OA [6]. However, only a small part of the population treats their complaints by participating in physical therapy or exercise therapy [7, 8]. The patients’ reasons for exercising (or not) depend on their (perception of their) physical ability for exercise; beliefs about exercise; motivational factors such as enjoyment, social support, taking control of the disability; pain and limitations of the lower limb [9].

Aquatic exercise enjoys a good reputation among patients because exercising in water feels easier and less painful than on land [10, 11]. The buoyancy of the water results in decompression of joints and causes the individual to feel weightless and to move more smoothly than on land [11, 12]. In addition, a warm water temperature promotes muscle relaxation, possibly resulting in pain reduction and the perception of less joint stiffness [12, 13]. Recent systematic reviews of aquatic exercise studies of individuals with OA and other chronic musculoskeletal disorders showed a small to moderate effect on joint pain, self-reported functioning, and performance tests of physical functioning [14, 15]. These achievements are comparable to the results of land-based training [16]. Growing recognition of the benefits of aquatic exercise and increasing public interest have resulted in many forms of aquatic exercise. Older patients with OA value individualized, expert-supervised shallow-water exercises, aqua jogging and hydrotherapy [17]. The exercise possibilities in water range from simple vertical water exercise and water running to more holistic programmes such as Watsu® and the adaptation of land-based fitness trends like Zumba® to the aquatic environment [18]. With the continual development and refinement of water-proof equipment, even spinning is now possible in a swimming pool. Aqua-cycling, where participants are immersed chest deep in water and pedal against water resistance, has recently become a popular water-based fitness activity. It combines the advantages of the aquatic environment with those of stationary land-based cycling, a combination that seems ideal for patients with knee OA. Stationary cycling is often used in the treatment of lower-limb injuries and chronic conditions like OA because of the reduced joint load, the repetitive circular pedalling movement that can be used to improve range of motion (ROM) in a functional manner, and the involvement of the largest muscle groups of the lower limb [10]. Evidence shows that stationary cycling can reduce knee pain and improve aerobic capacity, self-reported physical functioning and gait [19, 20]. So far, only a small number of studies have documented the therapeutic effects of aqua-cycling. Ulatkowski and von Kathen evaluated the additional effect of aqua-cycling during recovery from total knee surgery and anterior cruciate ligament reconstruction [21, 22]. In both cases, patients who did aqua-cycling showed greater improvements in knee-ROM and a reduction in knee joint swelling compared with patients receiving usual care only. Furthermore, a small one-group pre-test and post-test study on the effects of a 10-week aqua-cycling programme involving patients with rheumatic diseases showed a positive influence on strength, well-being and joint mobility [23]. Another small study on the feasibility of aqua-cycling, as a part of an aquatic circuit training for patients with knee OA, evaluated aqua-cycling as a safe and controlled exercise regimen and reported that participants were very satisfied with the training [24].

A 12-week group-based aqua-cycling training for mild to moderate knee OA patients was developed, because currently only a few therapeutic aqua-cycling interventions are available. The results of this study might provide guidance on the clinical use of aqua-cycling and greater insight into the effectiveness of aqua-cycling may help to broaden aquatic treatment possibilities. Furthermore, the study may support instructors of community aqua-cycling classes in dealing with participants with knee OA. For these reasons, it is important to examine whether a 12-week aqua-cycling programme, in supplement to usual care, will result in better outcomes of self-reported knee pain and physical functioning when compared with the relatively less intricate regimen usual care only.

This article provides a full description of the study’s rationale, design and method in accordance with the SPIRIT guidelines for reporting protocols of intervention trials and the CONSORT guidelines [25, 26].

## Method

### Study design

The current study is a single-blind, parallel-group, randomised controlled trial (RCT) of Maastricht University Medical Centre+ (MUMC^+^). Due to the structure of the trial, participant blinding is not possible. To design the trial as cost-effectively as possible, the programme coordinator is involved in many project activities such as recruitment, data collection planning and execution of the intervention, precluding blinding. Data collection and entry is performed by blinded and independent physical therapists and research assistants. The data will be analysed by blinded analysts.

The randomisation procedure is performed by an independent research assistant of the Department of Epidemiology of Maastricht University using free, internet-based software to generate the random allocation schedule (http://www.randomizer.org). A block randomisation with a constant block size of eight patients and an allocation ratio of 1:1 is used to keep sample sizes equal across the intervention and control group.

### Setting and participants

Participants were recruited in a hospital (MUMC^+^) in the Dutch province of Limburg. Patients were recruited form March 2013 until October 2015. The source population were patients diagnosed with mild to moderate knee OA. They were diagnosed by an orthopaedic surgeon or nurse practitioner, and the diagnosis was based on clinical symptoms and X-rays. Patients with an indication for conservative management of knee OA were offered the opportunity to participate in the present study. The orthopaedic specialist briefly explained the project and asked the patient for their agreement to share contact information (name and telephone number) with the programme coordinator. Research on participation in self-management programmes shows that the recommendation of a health professional influences the decision on whether to take part in a programme [27].

Non-participation had no consequences for further treatment.

#### Inclusion criteria

Eligible patients [1] rated knee pain between four and seven on a 10-point numeric pain rating scale, [2] had a Kellgren/Lawrence score between one and three, [3] were able to cycle on a stationary exercise bike, [4] were in good mental health (score <8 for anxiety and depression on the Hospital Anxiety and Depression Scale, HADS), [5] had sufficient language skills and [6] had an indication for physical therapy in conjunction with impairments due to OA.

#### Exclusion criteria

Potential participants with any contra-indication for aquatic exercise therapy such as [1] severe, unstable cardiorespiratory co-morbidities and [2] open wounds, or patients on a [3] waiting list for total knee surgery were excluded from participation in this study. Furthermore, all potential participants who met one of the following criteria were excluded given that these conditions could limit safe and full participation in the study or impede the perception of symptoms of knee OA: [4] corticosteroid injection <3 months and/or hyaluronic acid injection <6 months, [5] severe joint complaints (other than knee joint) that interfere the ability to participate in an exercise programme, [6] symptomatic and radiological apparent hip OA, [7] inflammatory joint diseases, [8] inability to safely enter and exit the pool and [9] fear of water.

Eligible patients first received verbal information by telephone. Interested patients were contacted by the programme coordinator after their consultation visit at the MUMC^+^. If their interest in participation continued after the telephone call, the programme coordinator sent additional information by mail. Each potential participant could consider participation for 1 week and was instructed to hold off any physical therapy until the randomisation results were known. If a candidate decided to participate, they had to sign an informed consent form in which they declared their voluntary participation. The programme coordinator checked incoming applications, including two short questionnaires, to screen for any contra-indications for physical activity using the physical activity readiness questionnaire (PAR-Q) and to screen for anxiety and depression using the HADS [28, 29]. In case of any doubts about a patient’s mental and/or physical health, the patient concerned was advised to contact a medical specialist for examination or advice.

**Fig. 1:**
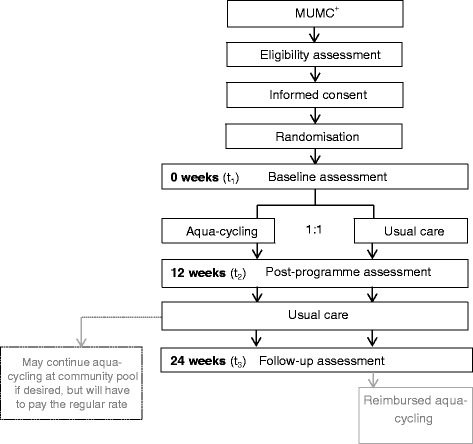
Participant timeline

After providing their informed consent, participants were randomly assigned to either the usual care control group or a 12-week aqua-cycling programme at MUMC^+^. Having completed the baseline assessment, participants in the intervention group started the aqua-cycling programme (24 sessions) and the control group could start with physical therapy and continue other usual care routines. The post-programme and follow-up measurements were scheduled after 12 and 24 weeks. After the last assessment, the control group is offered 12 sessions of aqua-cycling in a public swimming pool. The intervention group can also join this group after the 12-week intervention phase, but will have to pay the regular rate for aquatic fitness charged by the community pool. An overview of the participant timeline is given below in Fig. 1.

### Intervention

#### Both groups

Participants were referred by their general practitioner for a consultation visit to the orthopaedic specialist at MUMC^+^. Essentially, there were three different types of consultation visits. Participants from the area of Maastricht who have not yet been diagnosed with OA were referred to MUMC^+^ for further diagnostics of their knee complaints. Based on predictive values for severity of complaints, the MUMC^+^ scheduled patients for consultation at the Early OA Outpatient Clinic or the department of orthopaedic surgery. The Early OA Outpatient Clinic is responsible for the diagnosis and secondary prevention in patients with pre-surgical knee OA. The diagnosis is based on recent X-rays and clinical symptoms. Subsequently, the nurse practitioner provided patients with personalised information on OA, an information booklet on OA and an individual intervention plan consisting of lifestyle recommendations, medication routine and referral to a physical therapist. After 6 weeks, patients had their second consultation visit to evaluate the treatment. Patients already diagnosed with knee OA and who came back for a follow-up visit at the department of orthopaedic surgery could also participate in the study in case of an indication for physical therapy. These were usually patients who had limited success with pharmacological treatments such as injections and oral pain medication. In the case of injections, patients could still participate in the present trial after a wash-out period of three (in the case of a corticosteroid injection) to 6 months (in the case of a hyaluronic acid injection). If participants received an injection during the trial, the programme coordinator recorded the date and type. Occasionally, patients were referred to the orthopaedic surgeon for diagnosis and in that event the orthopaedic surgeon provided a diagnosis, lifestyle recommendations and a treatment plan.

All participants were instructed to maintain their usual care routine. The programme coordinator kept track of changes in participants’ treatment plans by monitoring patients with diaries that recorded OA-related functional problems, knee pain, physical activity, physical therapy and medication use in the first and third month after baseline assessment. Furthermore, a short interview by phone (control group) or in person (intervention group) was scheduled after 6 weeks. Prior to the last assessment, the programme coordinator called participants and inquired about any changes in treatment.

#### Control group

The control group was instructed to continue usual care, including working on prescribed lifestyle recommendations, medication routine and consultations with their orthopaedic surgeon during the 24-week trial. Furthermore, participants could start with physical therapy, but this was not necessary to participate in the present study. Use of and compliance with non-pharmacological interventions, such as physical therapy and exercise, is low in patients with knee OA [7]. Motivation to start with and maintain such interventions is influenced by previous treatment experience and perceived effectiveness, attitudes towards exercise, perceived severity of knee symptoms and comorbidity [30, 31]. In addition, due to differences in health care coverage, some participants were unable to afford physical therapy. Funding constraints made it impossible to cover the costs for these participants. To minimise dropout, participants were offered 12 weekly sessions of aqua-cycling after the end of a patient’s participation in this study. These sessions were held in a community pool because of size restrictions of the hospital pool.

#### Intervention group

Participants in the intervention group also continued with usual care, though they were instructed not to start additional physical therapy during the 12-week intervention period. Supervised by a physical therapist, participants performed aqua-cycling exercises for 45 min twice a week over a period of 12 weeks. The training took place in a heated therapy pool (32° Celsius) at the MUMC^+^ department of physical therapy. Depending on the body length of the participants, the water depth varied between 1.20 and 1.30 metres and participants were immersed between the navel and a maximum height of the xiphoid process (Additional file 1: Figure S1). The aqua bike used was the AquaCruiser II® from AquaKinetiqs (Additional file 2: Figure S2). This bike differs from other aqua bikes used for recreational sporting activities by healthy people (www.hydrorider.com). Differences consists of cycling barefooted instead of using water shoes, the AquaCruiser II® saddle is more comfortable, and the resistance can be adjusted during pedalling via a knob located below the handlebar instead of being set on land prior to the session. The resistance can be adjusted by six reproducible and equal increments by means of a magnetic braking system.

Participants cycled on the aqua bike throughout the whole session. Every session consisted of a warm-up, a conditioning phase and a cool-down. A detailed overview of the programme, reported according to the framework of Leeden et al., is provided in Table 1 [32].

**Table 1:** Aqua-cycling programme

Interventions goals (ICF):
- b710: mobility of joint functions
- b715: stability of joint functions
- b740: muscle endurance functions
- b760: control of voluntary movement functions
- b620: proprioceptive function
Exercises
1. Cycling at self-chosen rpm 2. Mobilisation of upper body
3. *60-rpm cycling* = participants focus on pedalling at a minimum of 60 rpm 4.*Lower leg exercises (1-2 exercises per session)* 4. 1one-leg pedalling 4.2 emphasis on upward or downward pedalling movement 4.3 out-of-the-saddle position: standing climb 4.4 out-of-the-saddle position: standing flat 5. *Arm exercises (1 exercise per session)* 5.1 shoulder abduction/adduction = arm lifts 5.2 shoulder transverse abduction/adduction = fly backs 5.3 shoulder flexion/extension = walking arms/ arm pendulum (one-sided) 5. 4 elbow flexion/extension = curl 5.5 shoulder flexion and extension = arm pendulum ➢ *arm exercises will be combined with different hand positions* (from less intense to more intense): sweeping on water surface, hand slicing sideways through the water (‘cutting’), ‘fisting’, cupped hands (‘scoop’), open hand (‘fan’)
6. Backward pedalling 7. Knee range of motion exercise = sitting on the aqua bike, feet out of the pedals, flexion and extension of unloaded knees 8. Calf and hamstring stretching
General information:
-Main focus is on correct aqua-cycling technique, i.e. cycling with a cadence of 60 rpm, a good alignment of the lower legs and an upright posture -Set-up: • Warm-up: exercise 1, 2 • Conditioning: exercise 3, 4, 5 • Cooling-down: exercise 1, 6, 7, 8 -Total programme duration: 12 weeks (2 sessions per week) -Frequency (exercise time/repetitions) and resting time: • Warm-up: 5–10 min • Conditioning: exercise 3: 5–8.20 min exercise 4: 4 sets of 30–45 seconds, 1 min resting exercise 5: 4 sets of 1 min (~20–40 repetitions) , 1 min resting • Cooling-down: 5–10 min -Intensity (conditioning): 11–13 Borg Scale/70% of maximum heart rate ((220-age) x 0.7)) -Progression: • exercise 3: weekly increase of 15–20 seconds in cycling time • exercise 4: pedalling resistance (after session 6, depending on performance of the exercise plus no signs of overload in ongoing and previous sessions) • exercise 5: hand position > length of lever arm > speed/small to big amplitude > increased surface area using aqua gloves or discs (depending on performance of the exercise plus no signs of overload in ongoing and previous sessions) -Training devices: • Timer • Borg Scale • Aqua bike ‘AquaCruiser II®’ • Aqua discs • Aqua gloves • Aqua dumbbells

*ICF* International Classification of Functioning, Disability, and Health; rpm = revolutions per minute

During the warm-up, the focus was on good posture and ergonomic pedalling obtained by activating core muscles, on good alignment of hip, knee and foot, and on rhythmic pedalling. Furthermore, the upper body was also activated to acclimatise the whole body to the aquatic environment.

In the conditioning phase, participants cycled for 25 to 30 min at a moderate intensity level and combined continuous cycling with exercises for the upper body. In addition, patients cycled in out-of-the-saddle positions, did one-leg pedalling or emphasised one part of the pedal movement (e.g. by actively pulling the pedals upwards). Therefore, the conditioning phase essentially consisted of three segments: continuous cycling at a minimum cadence of 60 revolutions per minute (rpm), upper body exercises and lower body exercises. The continuous cycling segment consisted of at least 5 min of cycling at a minimum pedalling cadence of 60 rpm. Exercise duration was increased by 15 to 20 s each week. Based on a conditioning phase of 25 min, this is an increase of 1 % per week, which is lower than the recommended weekly increase of 2.5 % as advocated by the American Geriatric Society [33]. This is deliberate, however, as the assumption is that aqua-cycling is more demanding than stationary cycling on land [24, 33]. Increased pedalling resistance was offered with caution and only if a participant was able to cycle continuously at 60 rpm without adverse reactions such as increased knee pain after the session, because increased workload results in increased knee load. This is in turn reported to be associated with worsening of knee pain [34]. The upper body exercises were used as an active break for the lower limbs as the pedalling tempo decreases with the focus shifted from the legs to the upper body. In addition, the upper body exercises enabled a varied exercise programme and prevented monotony which might have occurred with 45 min of purely cycling. The exercises were typical exercises used in aquatic fitness to strengthen arms (biceps, triceps), shoulders (rotators, flexors, extensors) and upper back (e.g. rhomboids, latissimus). A single repetition maximum as guidance for exercise intensity cannot be transposed to the aquatic environment. Characteristically, aquatic strength exercises are repeated 20 times and more [35]. Previous research has shown that this is an effective training method to increase muscular strength in chronic pain and OA patients [36–39]. Additionally, the high number of repetitions allows time to rehearse the exercise to promote execution using strong, powerful movements with good technique and full ROM [40]. Floating devices and drag equipment were used to increase resistance and to provide a varied exercise programme. The equipment used has not been sponsored by the manufacturers. The more exhausting upper-body exercise routine was followed by exercises focusing on the lower limbs. Patients cycled in a half-seated or standing position, emphasised the upwards and downwards pedalling movement and/or cycled with one leg. There is currently no evidence regarding the influence of different body positions in aqua-cycling on knee joint load. Consequently, the results of biomechanical studies of stationary cycling on land have guided the development of this exercise segment [41, 42]. Research on the difference between seated and standing uphill cycling shows an increased activation of monoarticular hip and knee extensors. However, to keep knee load as low as possible, standing positions should be limited during each session. During a land-based spinning class, out-of-saddle positions account for approximately 16 % (~8 min) of the session’s total time (50 min) [43]. In comparison, the time spent cycling in standing positions in the aqua-cycling programme was 5 to 8 % (2 to 4 min) of the total cycling time (~45 min).

The cool-down consisted of slowly cycling forward and backward, knee-ROM exercises and static stretching of the lower limbs to decrease the heart rate, prepare participants for the change of body position (e.g. from sitting on the exercise bike to standing position) and environment (the pool floor slowly comes up during the stretching exercises), and to reduces experienced muscle soreness.

The exercise intensity was moderate and was regulated by the patients themselves based on their perceived exertion using the BORG scale [44, 45]. In addition, heart rate is monitored by a Polar Ft 7, Wearlink® + Hybrid chest strap during each training session, and peak and average heart rates are protocolled. An average heart rate of 70-75 % of the maximum heart rate is desirable and recommended by exercise guidelines for OA [46, 47]. Furthermore, the supervising physical therapist assessed the quality of the performance by judging compensational movements, postural control, safe execution, and level of exertion (assessed by the talk test). In the event of any doubt about a participant’s health status, the physical therapist discontinued the training and referred the participant to their general practitioner.

### Outcome measures

The current study investigates the effect of aqua-cycling on impairments due to knee OA, such as knee pain, reduced physical functioning over the previous week and on the assessment day, and knee stiffness. It also seeks to make an overall assessment of disease severity and lower limb muscle strength compared with a control group receiving usual care.

Furthermore, the phenomenon of aqua-cycling is explored in a more general health context through evaluating the effect of aqua-cycling on functional capacity, physical activity level and quality of life. Psychological measures on self-efficacy and fear of movement are also assessed. Outcomes are assessed in person, but to keep the number of missing values as small as possible, all questionnaires will be sent by mail to any participants unable to come to the MUMC^+^. An overview of all measures and timing of assessment is given in Table 2.

**Table 2:**
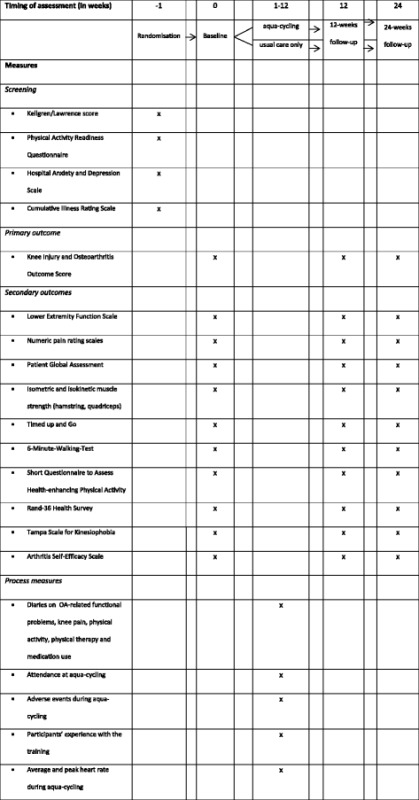
Overview of measures and timing of assessment

#### Primary outcome

The self-reported score on knee pain and physical functioning assessed with the *Knee Injury and Osteoarthritis Outcome Score* (KOOS*,*http://www.koos.nu) is the primary outcome measure. The KOOS questionnaire is an extended version of the Western Ontario and McMaster Universities Arthritis Index (WOMAC), which is a well-recognised, valid and responsible outcome measure in knee OA research [48]. In addition to the WOMAC subscales for pain, stiffness and physical function (in its complete and original format), the KOOS also takes into account difficulties with sport activities and knee-related quality of life. The five subscales are scored on a five-point Likert scale and final scores are modified to a 0–100 scale. A lower score is associated with higher impairments. The Dutch KOOS shows good, internal validity (Cronbach’s α: 0.71), construct validity (Spearman correlation between KOOS subscales and SF-36 pain and physical function: 0.63, 0.75) and is a reliable (ICC: 0.45-0.89) measurement for patients with mild to moderate knee OA [49]. The KOOS is self-administered and patients need approximately 10 min to answer all questions [50].

#### Secondary outcomes

The *Lower Extremity Function Scale (LEFS)* is a patient-reported measure on physical functioning on the test day [51]. The questionnaire consists of 20 questions and patients can complete it within a few minutes. The Dutch version of the LEFS has favourable psychometric properties: good internal consistency (0.96), reliability (ICC = 0.86) and a good construct and discriminant validity [52]. It is a disease-specific questionnaire and each item is scored on a five-point Likert scale. The total score ranges from 0 to 80 points. A higher score is associated with better physical functioning.

*Numeric pain rating scales (NPRS)* are frequently used to assess pain intensity in OA and the NPRS has been recommended as a core outcome measure for chronic pain trials [53, 54]. Previous research showed that the NPRS is a valid and responsive tool for pain measurements in OA patients and also a reliable tool (ICC: 0.64 to 0.86) in patients with orthopaedic problems and musculoskeletal pain [55, 56]. The NPRS is a self-administered scale, completed in less than 1 min and a lower score indicates less pain.

Osteoarthritis research societies have defined a core set of outcome measures for clinical OA trials: pain, function and *Patient Global Assessment* (PGA) [57]. Participants will be asked to consider all the ways in which illness and health conditions are affecting them at the time of the assessment and to mark one of 21 numbered circles on a visual analogue scale (VAS) [58]. A higher scores means that the participant feels more affected by their illness and health conditions. The PGA has a good test-retest reliability (ICC: 0.702) in patients with rheumatic arthritis and is completed by patients within a few seconds [58].

Data on *isometric and isokinetic muscle strength* of hamstring and quadriceps of the affected leg are collected with the dynamometer Biodex® System 3 Pro. The isometric quadriceps and hamstring muscle strength are tested in 30° and 60° fixation with three repetitions each. Isokinetic quadriceps and hamstring muscle strength are measured at 60° per second (five repetitions) and 180° degrees per second (five repetitions). The reliability of isometric and isokinetic strength testing is moderate (r = 0.8–0.9) to high (r > 0.9) in patients with mild knee OA [59].

The *Timed up and Go (TUG)* performance test measures the time needed by a patient to get out of a chair, walk three metres, return and get back into the chair. The guideline for physical therapy in patients with hip and knee OA of the Royal Dutch Society for Physical Therapy recommends the use of the TUG in combination with questionnaires (e.g. KOOS) to evaluate treatment goals for physical functioning [60, 61]. The inter-rater reliability between three physical therapists assessing patients with rheumatoid arthritis was high (ICC: 0.97) [62]. Intra-session reliability was also satisfactory: ICC of 0.75 with a time interval of more than 25 weeks and an ICC of 0.87 with a time interval of less than 1 week [63, 64]. In frail elderly patients and elderly patients undergoing orthopaedic rehabilitation, the TUG correlates well with gait speed (r = -0.61, 0.745) and performance of day-to-day activities (r = -0.78) and correlates highly with the Berg Balance Scale (r = -0.81) [65, 66].

The *6-Min-Walking-Test (6MWT)* is a simple test, recommended by the Dutch physical therapy guideline for OA, to assess functional capacity [61, 67]. Over a period of 6 min, participants walk at a self-chosen speed with the aim of covering as much ground as possible. Participants have to walk in a square with a total length of 44 metres. This set-up deviates from the standard as recommended by the American Thoracic Society which includes a 30 metre corridor or walkway with cones placed at the beginning and end of the 30-metre boundary to indicate turns [68]. In patients with fibromyalgia and those recovering from total hip and knee surgery, the 6MWT is a reliable test with an ICC for test-retest reliability of 0.94 and 0.98 [63, 69]. In terms of validity, the oxygen uptake during the 6MWT shows a high correlation with peak oxygen uptake values (r = 0.86) obtained during maximum exercise testing in patients with heart failure [70].

The *Short QUestionnaire to ASsess Health-enhancing physical activity (SQUASH)* is a survey to assess habitual physical activity and consists of eleven questions on physical activity in four different contexts: commuting, leisure time, during work and household activities. It is a short and simple questionnaire with proper reliability and validity [71]. The SQUASH is used to evaluate adherence to the Dutch physical activity guideline, recommending 30 min or more of at least moderate intense physical activity for a minimum of 5 days per week [72]. With regard to OA, only one study evaluated the SQUASH. Wagenmakers et al. found a good correlation with an accelerometer (r = 0.56) in patients with hip OA after surgery [73].

The *Rand 36-item Health Survey* (Rand-36) is a generic tool to measure health-related quality of life (HRQoL) [74]. It consists of 36 items that cover eight HRQoL domains: physical functioning, role limitations because of physical health problems, bodily pain, general health perception, vitality, social functioning, role limitations because of emotional problems, and mental health. The total score ranges from 0–100, with a higher score indicating better health status. The Rand-36 is almost identical to the Medical Outcome Study (MOS) Short-Form-36 (SF-36), and both have a proven sound responsiveness in patients with knee OA (SRM = 0.528), and internal consistency (Cronbach’s α: > 0.70) and test-retest reliability (ICC: 0.40-0.82) in a Dutch general population [75–79].

The *Tampa Scale for Kinesiophobia (TSK)* is used to assess fear of injury/re-injury due to movement [80]. It is a 17-item scale that is scored on a four-point scale from ‘strongly disagree’ to ‘strongly agree’. The present study uses the Dutch version which shows good psychometric properties in patients with acute low back pain: good internal consistency (Cronbach’s alpha = 0.70) and satisfactory test-retest reliability (ICC: 0.76) [81].

The *Arthritis Self-Efficacy Scale (ASES)* is a valid and responsible measure providing information on patients’ self-efficacy to perform a task (e.g. ‘How certain are you that you can walk 100 feet on flat ground in 20 seconds?’) or to achieve a specific behaviour (e.g. ‘How certain are you that you can decrease your pain quite a bit?’) [82, 83]. In total, the scale consists of 20 items that are divided into three subscales: self-efficacy pain scale, self-efficacy function scale and self-efficacy other symptoms scale (e.g. fatigues, enjoyment). The items are scored on a ten-point Likert scale resulting in a total score ranging from 0 to 100. Higher scores indicate a better self-efficacy. The ASES has been translated and is available in Dutch [84]. The present study measures the self-efficacy for function. This subscale has a good test-retest reliability (ICC: 0.85) and internal consistency (Cronbach’s alpha = 0.89) [85].

#### Process measures

*Daily diaries* collect information on knee pain, physical functioning, level of physical activity, pain medication routine and physical therapy participation over two 30-day periods (during the intervention period). Participants can fill in the diaries on a computer or in a printed booklet version. Information on physical functioning and physical activity is gathered by questions derived from the LEFS and SQUASH questionnaires [52, 72]. Knee pain is measured by NPRS [55]. The section on medication use asks if pain medication is used for knee pain or other pain, the name of the pain medication and the dosage and time-point(s) of taking the medication. Participation in, duration and intensity of exercise routines or physical therapy will be documented as well. Furthermore, four questions, derived from the RAND-36 questionnaire, will ask about the restrictions in physical role functioning. Previous research only shed light on the level of hindrance and/or avoidance of activities, but not on the type of hindrance [86]. The daily repeated measures will provide more insight into the course of pain, physical functioning, physical activity and medication use. The diary data from the intervention group will yield important information on the development of impairments, level of physical activity and medication use in response to the aqua-cycling programme. The diaries of the control group will provide a picture of the level of physical activity, participation in exercise therapy and the development of impairments over time. Previous research using booklet diaries and comparable diary periods showed good compliance and a low dropout rate, indicating that this method is acceptable for chronic pain patients [87].

*Participants’ experience with aqua-cycling* will be assessed after the final training session by means of planned focus-group sessions. Small-group interviews will be planned, and participants will be asked broad, open-ended questions about their expectations, fulfilment of expectations, positive and negative aspects of the training and suggestions for further development of the training. Thirty per cent of the participants (~20 participants) in the intervention group will be invited to attend small-group interviews to provide feedback.

*Attendance, adverse events and exercise progression of the intervention group * were registered by the physical therapist. For every patient a training log book exists where the physical therapist documented date and number of the training sessions attended. In total every patient could attend 24 sessions. Pedalling tempo and resistance, heart rate and BORG scores were noted for every exercise during the conditioning phase. Furthermore, the physical therapist documented the occurrence and type of problems with the performance of certain exercises in an indicated open text box in the training logbook. Thus, the physical therapist described the type of problem, whether the participants were able to continue the exercise and in case of performance restrictions the alternative exercise was described. Also, adverse effects during or following the sessions were documented in the training log book. Non-serious adverse effects were defined as increased joint pain, stiffness, muscle soreness and/or fatigue occurring during or immediately after the last training session [88, 89]. If these adverse events were experienced longer than 24-h or interfered with physical activities and social participation they were classified as severe adverse events [89]. A serious adverse event was defined as an occurrence that resulted in permanent or severe disability, hospitalization, or death [90].

### Data collection and management

The data from all measurements will be recorded on paper by the blinded outcome assessors. Patients are instructed not to inform the assessor about group allocation.

The outcome assessors are physical therapists of the MUMC^+^. The performance measures and strength assessments are part of their routine tasks and no special training prior to the study was needed. The purpose and scoring method of all questionnaires used was explained by the programme coordinator prior to the start of the study. Furthermore, the outcome assessors practised data collection several times in order to get an idea of the time needed. The data are recorded on paper, with numbers used to represent the rank order within the recruitment process in order to guarantee that the data is analysed separately from personal data. The data in these paper case reports are digitised by research assistants and the programme coordinator will enforce data integrity through range checks and cross-validation between the same variables assessed on repeated occasions. In addition, visual record verification will be done by comparing the first ten records of a data set with the corresponding paper case reports [91]. If no inconsistency is found, the programme coordinator will check every tenth record until an incorrect record is found. After correction of the incorrect record, all following records will be checked until successive records free of inconsistencies are found [92]. All data on paper will be stored in a locked archive for a maximum of 15 years. Only the programme coordinator has access to personal data. After the analysis, other researchers of the team (RAB, IM, AFL, and PJE) will also have access to anonymous data.

### Sample size

The present study is the first to evaluate the effects of aqua-cycling in patients with mild to moderate knee OA. There are no previous data on which to base the sample size calculation. The estimation of the sample size is based on two factors: [1] the minimum clinically important difference (MCID) of WOMAC, and [2] studies with a similar design (aquatic therapy versus usual care) or intervention (one group pre-test/post-test feasibility study of aqua-cycling for rheumatic patients). Although the present study uses the KOOS questionnaire, the WOMAC questionnaire has been used to estimate the sample size. The WOMAC is well recognized in OA research and the questionnaire and minimum clinically important differences (MCID) of the WOMAC subscales are known. The MCID changes from baseline to post-interventions on the WOMAC pain and function scale range from 15 % to 18 % for pain and 12 % to 17 % for physical function [75, 93]. The results of previous studies are in line with or exceed the MCIDs referred to. Hinman et al. showed a 21 % and 29 % improvement in WOMAC pain and function scores for the hydrotherapy group [36]. The usual care group did not improve. A feasibility study of an aqua-cycling programme for rheumatic patients showed an improvement of 14 % in the post-intervention score of self-reported physical functioning [23]. Based on the above mentioned data, the aqua-cycling training in the present study is expected to achieve at least similar results as the interventions of Moser and Hinman, or even exceed those results because of a higher exercise frequency and intensity and longer duration of the intervention [23, 36]. Thus, a difference of 25 % between the aqua-cycling group and usual care group in terms of reduction of knee pain and improvement in physical functioning is hypothesised as clinically meaningful. The statistical level of significance was set to an alpha (α) of 0.05 and statistical power to 0.80. The standard deviation is 20 % of the maximum score of the WOMAC subscale for pain and physical function [94]. With an expected dropout rate of 20 %, the final number of participants needed is 168.

### Data analysis

Data analysis will be performed using IBM SPSS Statistics 23. The effect of group membership (aqua-cycling versus usual care) on primary and secondary outcomes will be estimated and tested for significance with a significance level set at 0.05. Furthermore, any significant changes that occur over time will be examined. Demographic variables and clinical background variables (i.e. BMI, co-morbidities) will be used as grouping variables for subgroup analysis or as covariates.

Multilevel analysis will be applied with repeated measures (level 1) that are clustered within persons (level 2), and with patients (level 1) clustered within groups (level 2). Using multilevel analysis allows the use of all data available, including dropout, loss to follow-up, missed appointments and participant incapacity.

Diary data will be examined for the time course of level of physical activity, physical functioning, knee pain and pain medication use in the intervention and control group, and for between-group differences in change. In addition, the relationship between the aqua-cycling training and the factors just referred to will be evaluated. Multilevel analysis will be used to estimate and test between-person differences and the within-person processes.

### Data monitoring

The content of the aqua-cycling intervention is comparable to existing physical activity programmes on land. Research has shown that these programmes involve no additional harm or risk to the patient [47, 95]. Aqua-cycling in rheumatic patients was evaluated in a study as safe and feasible [23]. In addition, there is adequate evidence that aquatic training and stationary cycling are beneficial and safe activities for patients with knee OA [14, 19, 20].

Because the risk of any adverse events from participation in the intervention group is small and comparable to the very low risk of adverse events from participation in land-based OA exercise programmes [47], no data monitoring committee (DMC) is needed. In case of a serious adverse event, the programme coordinator will inform all professionals involved in the study and report the event via a web portal to the accredited Medical Ethics Board within 24 h.

### Ethics

Ethical approval has been obtained from the Medical Ethics Board of MUMC^+^ (reference number 12-2-075) on 06-03-2013. The trial was registered on 21-12-2012 in the Netherlands Trial Register (NTR3766). Any modifications to the protocol that influence the execution of the trial or participant safety, i.e. changes of study design or procedures will be described in a formal amendment. All substantial amendments will require approval from the Medical Ethics Board of MUMC^+^.

Participants in the study are covered by an insurance policy that includes cover against research subject injury or death as a result of the study. The research project is covered by liability insurance which is in accordance with Section 7, subsection 6 of the Medical Research (Human Subjects) Act (WMO). A copy of the insurance certificate of MUMC^+^ is in the possession of the board of the Medical Ethics Committee.

## Discussion

This trial will demonstrate if the newly designed aqua-cycling intervention, in supplement to usual care, can help to improve impairments due to knee OA. As far as we know, the present study is the first randomised controlled trial evaluating the effects of aqua-cycling in the pre-surgical stage of OA. If this training proves to be effective, the results can provide guidance on the use of aqua-cycling in clinical and community exercise settings. Aqua-cycling could be used to increase the range of motion of the knee, lower limb muscle strength and aerobic capacity in all populations, whereas land-based training is too painful. It might also an option for patients who feel uncomfortable with traditional aquatic exercise because of poor swimming skills or hydrophobia. Previous studies have shown that aqua-cycling is well accepted by patients who have hydrophobia [22, 24]. As aqua-cycling has become a recent fitness trend in Europe and the US, many public swimming pools offer aqua-spinning to a healthy population. Exercise instructors in community exercise settings who are qualified to supervise classes with musculoskeletal disorders could use the training programme described (if proven effective) to adapt aqua-spinning classes to the needs of people with knee OA. The opportunity to participate in a modern and popular exercise class might especially be appealing to knee OA patients who want to be active and/or are young [20].

The strength of this study is the close monitoring conducted during the intervention phase with diaries, since self-reported measures might be sensitive to day-to-day variations not capturing the development of OA impairments throughout the intervention [19]. Another strong aspect of the study is the follow-up assessment 3 months after the end of the programme, something that is rarely done in aquatic exercise research [14]. Especially interesting in the follow-up assessment is the evaluation of whether participants in the intervention group continued to aqua-cycle in the community swimming pool or stayed active in another way. This will indicate if people are willing to continue aqua-cycling at their own cost or if it has helped them to become more active. The control group will be invited to attend 12 free aqua-cycling sessions in the community pool. Due to limited access to the hospital pool and the limited number of aqua bikes (n = 4), it is not possible to train both groups in the hospital. Funding restraints make it impossible to bear the costs of 24 sessions twice a week for the control group in the community pool. As the programme coordinator will give the training, there will be no difference in terms of the training content and structure. However, this waiting-list control design can influence the results of the study in two ways. On the one hand, participants in the control group might be more motivated to follow usual care instructions with regard to physical activity because they do not want to be less active than the intervention group. In addition, this group will be monitored by means of diaries too, which might also motivate them to be more active. On the other hand, it is possible that the control group participants will follow usual care recommendations less strictly as they will be waiting for their turn to try out aqua-cycling. Furthermore, the fact the control group participants did not receive any immediate and free intervention might influence motivation for further participation. Therefore, we will inform participants about group allocation before the baseline assessment. The assessments of the present study are not part of the clinical routine and participants have to come back for the assessments after giving consent and being randomised. By informing participants about group allocation prior to the baseline, we wish to prevent frustration about group allocation and possible dropout. Nevertheless, this strategy increases the risk of dropout before baseline assessment.

In conclusion, this trial will increase the knowledge of aqua-cycling and might be a useful addition to aquatic exercise training. As with aquatic treadmill training, it is possible to adequately monitor and modify exercise intensity since pedalling rate and resistance can be adjusted [96]. In addition, the exercise programme is based on exercise guidelines and the exercise intensity will be measured by pedalling frequency and resistance, average heart rate, peak heart rate and perceived exertion during the different parts of the conditioning phase. Recent reviews strongly recommend using and reporting exercise intensity, as will be done in this study, in order to obtain a better understanding of the dose-response relationship in aquatic exercise [14, 15].

### Trial status

The data collection is still ongoing and will be completed in March 2016.

### Dissemination policy

The scientific integrity of this research project requires that all results of this study be disclosed unreservedly. The results will be submitted for publication to peer-reviewed scientific journals. Furthermore, the results will be presented at national and international congresses. Through to November 2016, five articles have to be submitted with the programme coordinator as first author. These articles will provide the basis for the programme coordinator’s PhD thesis. All authors must contribute significantly to the conception of an article and/or the analysis or interpretation of data. Each author needs to revise the concepts of an article critically and has to give final approval of the manuscript that will be published. It is not the intention to collaborate with professional writers.

The outcomes of the study will be released to the referring orthopaedic nurse practitioner and orthopaedic surgeons, the participating physical therapists, the local community swimming pool and the general medical community. In addition to the study results, every participant will receive an individual summary of her/his study results as soon as possible after participation.

## Additional files

Additional file 1: Figure S1. Basic position on the aqua bike (The subject provided consent for her image to appear in this figure). (JPG 900 kb) Additional file 2: Figure S2. AquaCruiser II®. (JPG 33 kb)
