# Supplementary figures and images for: Effect of aqua-cycling on pain and physical functioning compared with usual care in patients with knee osteoarthritis: study protocol of a randomised controlled trial
Source: BMC Musculoskelet Disord. 2016 Feb 18;17:88. doi: 10.1186/s12891-016-0939-5 (PMC4758142; doi:10.1186/s12891-016-0939-5)

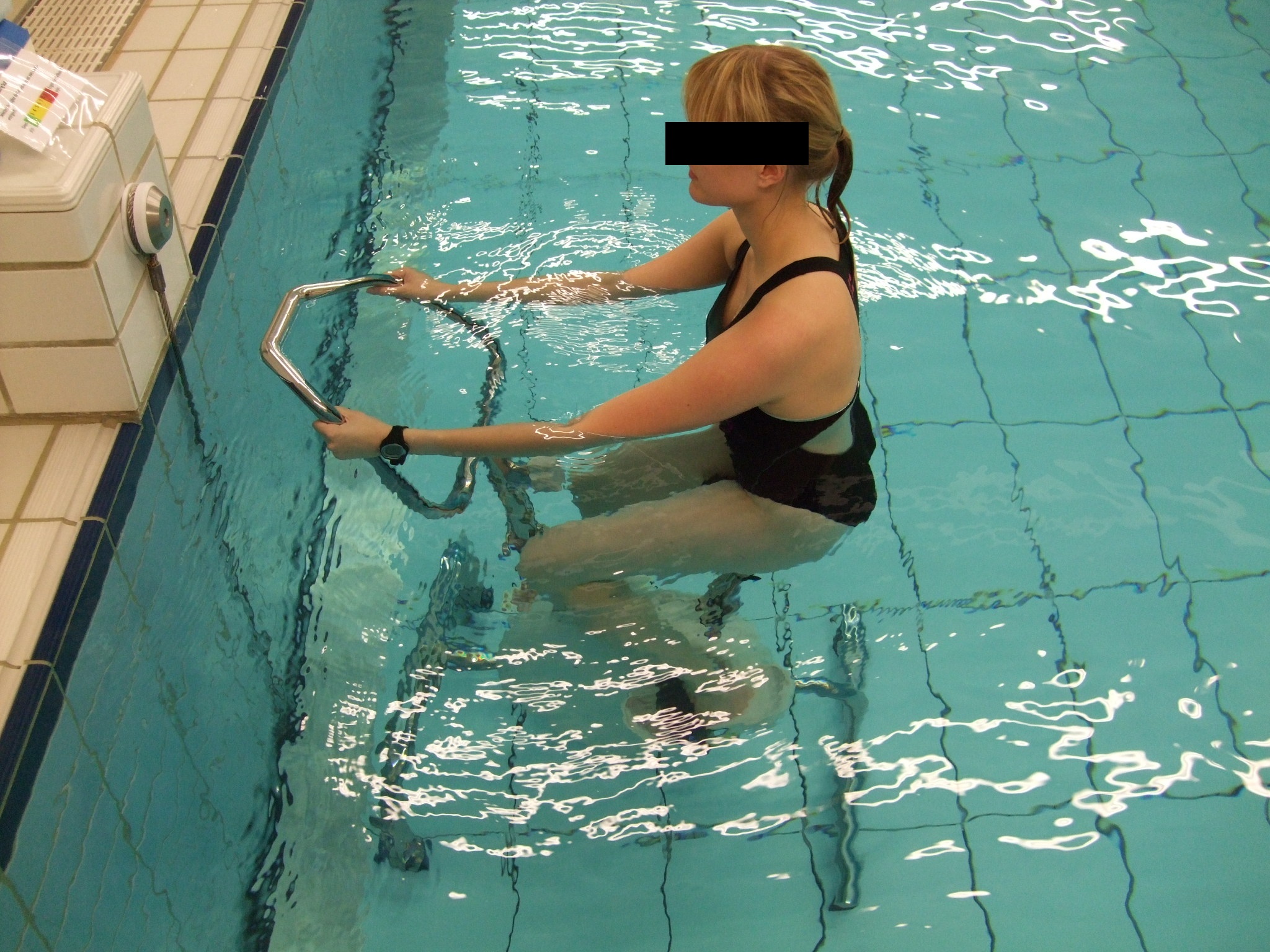

Supplement: Additional file 1: Figure S1. — Basic position on the aqua bike (The subject provided consent for her image to appear in this figure). (JPG 900 kb) [file 12891_2016_939_MOESM1_ESM.jpg]

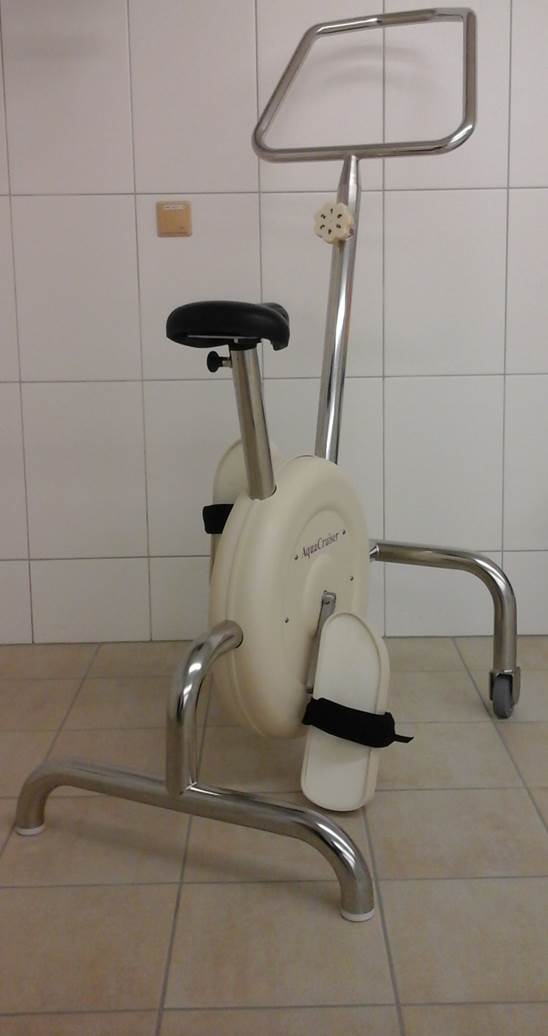

Supplement: Additional file 2: Figure S2. — AquaCruiser II®. (JPG 33 kb) [file 12891_2016_939_MOESM2_ESM.jpg]
